# Identification of Maltase Glucoamylase as a Biomarker of Acute Kidney Injury in Patients with Cirrhosis

**DOI:** 10.1155/2019/5912804

**Published:** 2019-04-16

**Authors:** Linda Awdishu, Shirley Tsunoda, Michelle Pearlman, Chanthel Kokoy-Mondragon, Majid Ghassemian, Robert K. Naviaux, Heather M. Patton, Ravindra L. Mehta, Bhavya Vijay, Satish P. RamachandraRao

**Affiliations:** ^1^UC San Diego Skaggs School of Pharmacy and Pharmaceutical Sciences, San Diego, USA; ^2^Biomarkers Laboratory, O'Brien Center for Acute Kidney Injury Research, Nephrology-Hypertension, UC San Diego, Department of Medicine, San Diego, USA; ^3^UC San Diego, Department of Medicine, Division of Gastroenterology, San Diego, USA; ^4^UC San Diego, Department of Chemistry & Biochemistry, Biomolecular & Proteomics Spectrometry Facility, San Diego, USA; ^5^UC San Diego, Departments of Medicine, Pediatrics and Pathology, San Diego, USA; ^6^I-AIM Biomarkers Laboratory, The University of Trans-Disciplinary Health Sciences and Technology (TDU), Bangalore, India; ^7^UC San Diego, Department of Medicine, Division of Infectious Diseases, San Diego, USA

## Abstract

**Background:**

Acute kidney injury (AKI) is a frequent complication of decompensated cirrhosis with increased mortality. Traditional biomarkers such as serum creatinine are not sensitive for detecting injury without functional change. We hypothesize that urinary exosomes potentially carry markers that differentiate the type of kidney injury in cirrhotic patients.

**Methods:**

This is a prospective, single-center, and observational study of adult patients with cirrhosis. The patient groups included healthy normal controls, compensated cirrhosis with normal kidney function, decompensated cirrhosis with normal kidney function, and decompensated cirrhosis with AKI. Data were extracted from the electronic health record including etiology of liver disease, MELD score, history of decompensation, Child-Turcotte-Pugh score, history of AKI, and medication exposures. Urine samples were collected at the time of consent. Urine exosome protein content was analyzed, and proteomic data were validated by immunoblotting. Statistical analysis included partial least squares-discriminant analysis coupled with variable importance in projection identification.

**Results:**

Eighteen cirrhotic subjects were enrolled, and six healthy control subjects were extracted from our biorepository. Urine exosomes were isolated, and 1572 proteins were identified. Maltase-glucoamylase was the top discriminating protein confirmed by western blotting.

**Conclusions:**

Patients with cirrhosis and AKI have upregulation of renal brush border disaccharidase, MGAM, in urinary exosomes which may differentiate the type of kidney injury in cirrhosis; however, the clinical significance of this requires further validation.

## 1. Introduction

Acute kidney injury (AKI) occurs in approximately 20% of hospitalized patients with cirrhosis [1, 2]. AKI in hospitalized cirrhotic patients is frequently progressive, severe, and an independent negative predictor of mortality [3]. The most common cause of AKI in cirrhosis is hemodynamic, accounting for 70% of cases. Acute tubular necrosis (ATN) accounts for 30% of cases, and postrenal causes are rare accounting for less than 1% of cases. Hepatorenal syndrome (HRS) is hemodynamic without an identifiable kidney injury or disease and occurs in approximately 20% of cirrhosis patients [4, 5].

Serum creatinine (Scr) is the most widely used biomarker to assess kidney function and identify kidney injury. However, Scr is suboptimal in cirrhotic patients for numerous reasons including decreased liver production, muscle wasting with diminished stores, increased volume of distribution, and protein calorie malnutrition. Average Scr values are lower in cirrhotic patients compared to the general population, resulting in delayed diagnosis of AKI based on the current definition of AKI [6]. Additionally, Scr is a biomarker of kidney function and is not a sensitive injury marker. Novel biomarkers of kidney injury have emerged to improve detection of AKI and aid in differentiating the etiology of AKI. Kidney damage biomarkers including kidney injury molecule-1 (KIM-1), neutrophil gelatinase-associated lipocalin (NGAL), interleukin-18, liver fatty acid binding protein (L-FABP), insulin like growth factor binding protein-7 (IGFBP-7), and tissue inhibitor of metalloproteinase-2 (TIMP-2) may be elevated prior to an increase in Scr enhancing detection of kidney damage without functional change [7]. Studies have demonstrated that these biomarkers may differentiate the etiology of AKI [8, 9]. Improved biomarkers for detecting and differentiating AKI represent important unmet clinical need in patients with cirrhosis.

Exosomes are nanovesicles that are released from living cells as a mechanism of intercellular communication [10]. The protein content of exosomes has been shown to be remarkably modified under pathological or stress conditions [11–13]. In the kidney, exosomes are delivered to urine from all cell types [14, 15], and urinary exosomes can potentially be considered as the biochemical signature of the subject. Since urinary exosomes are not routinely assayed, they may provide additional unrecognized information on protein biomarkers of AKI in patients with cirrhosis.

The aim of this study was to evaluate urinary exosome proteomics in patients with cirrhosis and AKI compared to healthy subjects. We hypothesized that the urinary exosome protein content differs in patients with compensated or decompensated cirrhosis experiencing AKI compared to normal healthy control subjects. Furthermore, we postulated that differential urinary exosomal protein content would offer insight into mechanisms of kidney injury in cirrhosis.

## 2. Materials and Methods

This is a prospective, single-center, and observational study of adult patients with cirrhosis. All patients for the study were recruited from the UC San Diego Health system between July 1, 2013, and June 1, 2014, and provided informed consent. Patients were eligible for inclusion if they had a diagnosis of cirrhosis and were able to provide a urine sample. Cirrhosis was determined by liver biopsy, cross-sectional imaging, or clinically (via identification of a decompensation event as determined by a hepatologist). Data were extracted from electronic health records including demographics, anthropometrics, vital signs, comorbid medical problems, etiology of cirrhosis, complications of cirrhosis (ascites, varices, hepatic encephalopathy, and hepatocellular carcinoma), history of AKI or chronic kidney disease, and medication exposures within 30 days of enrollment. Only patients with complete clinical data and laboratory tests within 30 days of enrollment were eligible for inclusion in this study. Patients were categorized into groups as follows:

1. Group 0: normal healthy controls
2. Group 1: compensated cirrhosis (Child-Turcotte-Pugh class A, MELD <10) with no history of AKI and normal kidney function
3. Group 2: decompensated cirrhosis (Child-Turcotte-Pugh class B or C) with no history of AKI and normal kidney function
4. Group 3: decompensated cirrhosis (Child-Turcotte-Pugh class B or C) and AKI

Normal kidney function was defined as an estimated GFR > 60 ml/min/1.73 m^2^ (MDRD formula), no albuminuria and no history of AKI. AKI was defined according to AKIN criteria: Scr rise of 0.3 mg/dl in 48 hr or 50% rise in Scr from baseline [16]. Patients with AKI were recruited during admissions and consultations from the inpatient hepatology service if they had blood and urine specimens obtained during the episode of AKI. A fourth group of healthy controls was extracted from a healthy normal biorepository at the UCSD O'Brien Center for AKI Research at the UC San Diego School of Medicine. This work was approved by the Institutional Review Board of the University of California, San Diego.

### 2.1. Urine Sampling and Processing for Exosome Isolation

Urine was centrifuged at 3000 ×g for 30 min. The supernatant pH was adjusted to 7, aliquoted and frozen at −80°C. Exosomes were prepared using polyethylene glycol- (PEG-) induced precipitation [17]. The PEG-mixed urine samples were kept at room temperature for 2 hours and spun at 10,000 ×g for 30 minutes. The pellet was resuspended in 10 mM Tris with 1 mM EDTA-Na salt. This step was repeated twice to remove impurities. One-dimensional SDS-PAGE of the exosome proteins was conducted prior to in-gel trypsinization to prevent confounding [18].

### 2.2. Proteomic Analysis

The gel was cut to 1 mm × 1 mm and destained 3 times using 100 *µ*L of 100 mM ammonium bicarbonate for 15 minutes, followed by 100 *µ*L of acetonitrile (ACN) for 15 minutes [19]. The supernatant was lyophilized, and the resulting pellet was reduced with 200 *µ*L of 100 mM ammonium bicarbonate-10 mM DTT and incubated at 56°C for 30 minutes. After removing the liquid, gel pieces were added to 200 *µ*L of 100 mM ammonium bicarbonate-55 mM iodoacetamide. This was incubated at room temperature for 20 minutes in the dark. The supernatant was removed and washed with 100 mM ammonium bicarbonate for 15 min. Then, 100 *µ*L of ACN was added to dehydrate the gel pieces, and the solution was lyophilized. Ice-cold trypsin (0.01 *µ*g/*µ*L) in 50 mM ammonium bicarbonate solution was then added to cover the gel pieces for the digestion process and set on ice for 30 minutes. Once rehydration was complete, fresh 50 mM ammonium bicarbonate was added to replace excess trypsin and left overnight at 37°C. Extraction of the peptides was done twice by the addition of 50 *µ*l of 0.2% formic acid and 5% ACN and vortexed for 30 minutes at room temperature. After removing the supernatant, 50 *µ*l of 50% ACN-0.2% formic acid was added to the sample, vortexed again for 30 minutes at room temperature. This supernatant was removed and combined with the previous supernatant from the first extraction. Samples were analyzed using Eksigent nano-LC-Ultra® 2D with cHiPLC-nanoflex system (Eksigent, AB SCIEX Dublin, CA, USA) in a trap-elute mode in combination with tandem mass spectroscopy using the QExactive mass spectrometer (Thermo Fisher Scientific, San José, CA, USA) with electrospray ionization [17].

### 2.3. Data Management

The SEQUEST search engine (Thermo Scientific Proteome Discoverer software, version 1.4) was used in the analysis. The protein database for tryptic peptide sequences for *Homo sapiens* from the National Center for Biotechnology Information (NCBI) was used to compare our experimental MS/MS spectra. To identify peptide sequences and related proteins, we used previously published criteria [17]. To assess statistical significance, separate target and decoy searches and calculation of classical score-based false discovery rates (FDRs) were used. Finally, we filtered the SEQUEST output data to assign a final score to proteins. Minimum values of correlation score (Xcorr) of 1.5, 2.0, 2.25, and 2.5 were chosen for single-, double-, triple-, and quadrupole-charged ions, respectively. Previously published parameters were utilized to guarantee a high stringency [20], and the false-positive peptide ratio was less than 3%.

### 2.4. Statistical Analysis

The normalized spectral abundance factor (NSAF) was used to calculate the relative abundance of polypeptides [21]. Log transformation and scaling of peptide counts were performed prior to statistical analysis. MetaboAnalyst 2.0 web portal (http://www.metaboanalyst.ca) was used to perform Student's *t*-test, partial least squares discriminant analysis (PLS-DA), and variable importance in projection (VIP) with an a priori *p* < 0.05 [22]. The ratio of individual protein to total concentration was evaluated using the paired Student's *t*-test for each group. PLS-DA and VIP were used to identify discriminatory proteins [23]. We selected proteins with a false discovery rate (FDR) of ≤10% to validate using western blotting.

### 2.5. Western Immunoblotting and Quantification

The antibody against MGAM was purchased from Proteintech Group, Inc., (Chicago, IL, USA) and was used to resolve 100 *μ*g of protein from urine exosomes of each subject. After separation, the proteins were transferred to nitrocellulose paper, blocked, incubated with primary antibody overnight before washing with Tris-buffered saline, incubation for 1 h with HRP-secondary antibody conjugate, and visualized by developing as described in previous publications from our laboratory [24, 25]. ImageJ software (NIH) was used to quantify western immunoblot bands [24] and plotted (GraphPad Prism, San Diego, CA, USA).

## 3. Results

### 3.1. Clinical Characteristics of Cirrhosis and Healthy Control Subjects

Six patients in each group with complete clinical data were analyzed and compared to six healthy controls. Demographics and etiology of liver disease are summarized in Table 1. As anticipated according to study design, Child-Turcotte-Pugh and MELD scores varied significantly between patient groups.

### 3.2. Proteomic Analyses of Urine Exosomes from Cirrhotic Patients and Healthy Controls

**Figure 1:**
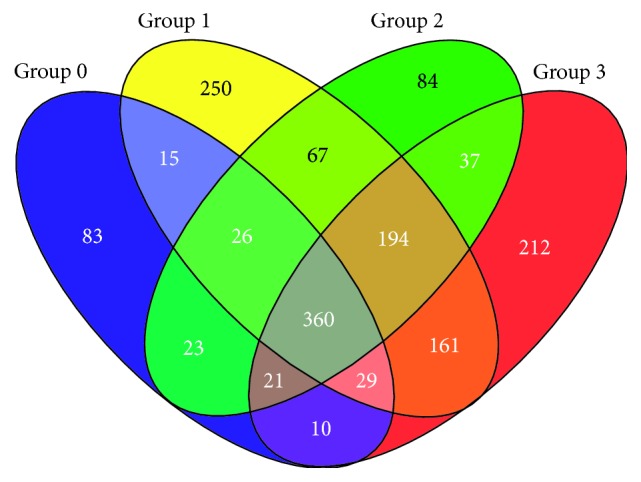
4-way Venn diagram depicting the distribution of proteins isolated from urinary exosomes of healthy controls (group 0), patients with compensated cirrhosis (group 1), with decompensated cirrhosis (group 2), and with decompensated cirrhosis and kidney injury (group 3).

**Figure 2:**
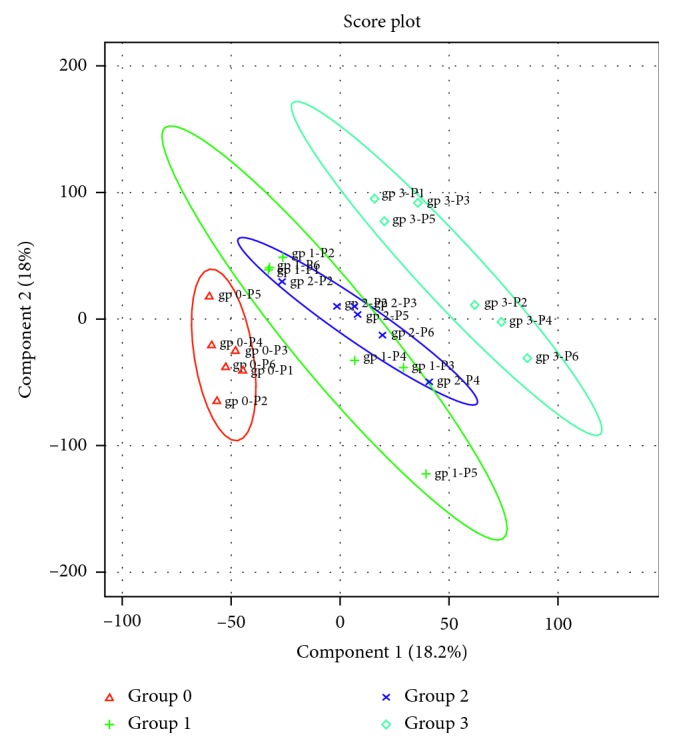
Two-dimensional (2D) partial least squares discriminant analysis separation using protein normalized spectral abundancy factor count-based proteomic measurements in the urine exosome of healthy controls (group 0), patients with compensated cirrhosis (group 1), with decompensated cirrhosis (group 2), and with decompensated cirrhosis and kidney injury (group 3). Clear separation of urine exosome proteins for control versus cirrhotics is observed, signified by the lack of overlap between the two groups of exosome proteins. However, among the cirrhotic subjects, the observed overlap between groups 1 and 2 may be due to crowding, as the expansion of the axes magnitude showed sufficient delineation.

In total, across all 4 groups, 1572 unique proteins were identified. There were 360 proteins that were common to all groups. We found 83 unique exosomal proteins for group 0 (controls), 250 for group 1, 84 for group 2, and 212 for group 3 (Figure 1). We further conducted multivariate PLS-DA on proteins, which showed clear separation between healthy control group and different subgroups of cirrhotic subjects, as shown in Figure 2. Compensated and decompensated cirrhotic subjects without kidney injury (groups 1 and 2) showed considerable overlap while cirrhotic subjects with AKI (group 3) showed clear separation from the other cirrhotic subjects and healthy control subjects. A separate ANOVA of proteins between the four groups showed that 126 proteins were significantly altered (*p* < 0.05), of which 13 reached the false discovery rate (FDR) cutoff of <10% (Table 2). Maltase-glucoamylase (MGAM) was the top discriminant protein with a VIP score of 4.35 for the entire study cohort.

### 3.3. Maltase-Glucoamylase Protein Is Increased in Decompensated Cirrhotic Urine Exosomes

**Figure 3:**
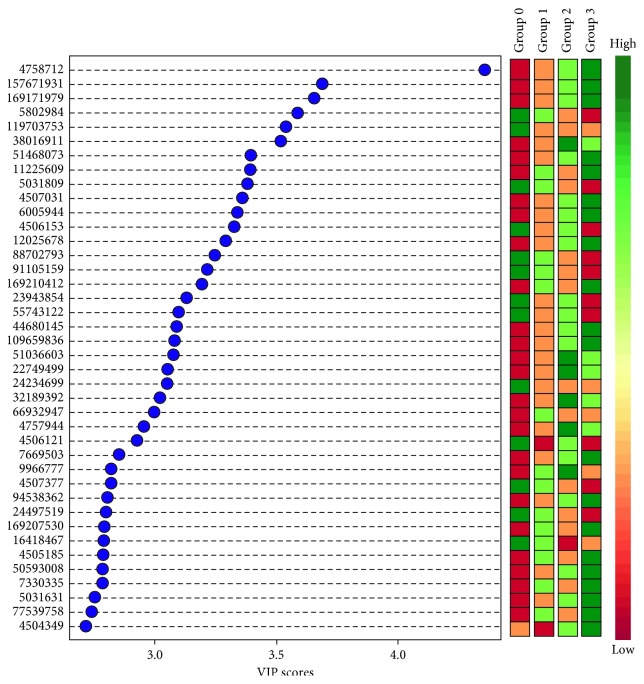
Variable importance in projection (VIP) plot: important features (analyzed NSAF scores of the proteins) identified by PLS-DA in a descending order of importance. The graph represents relative contribution of proteins to the variance between the cirrhotic subject and noncirrhotic subject control urine exosomes. High value of the VIP score indicates great contribution of the proteins to the group separation. The green and red boxes on the right indicate whether the protein concentration is increased (green) or decreased (red) in the exosome of the cirrhotic subject urine vs. noncirrhotic subject urine samples.

**Figure 4:**
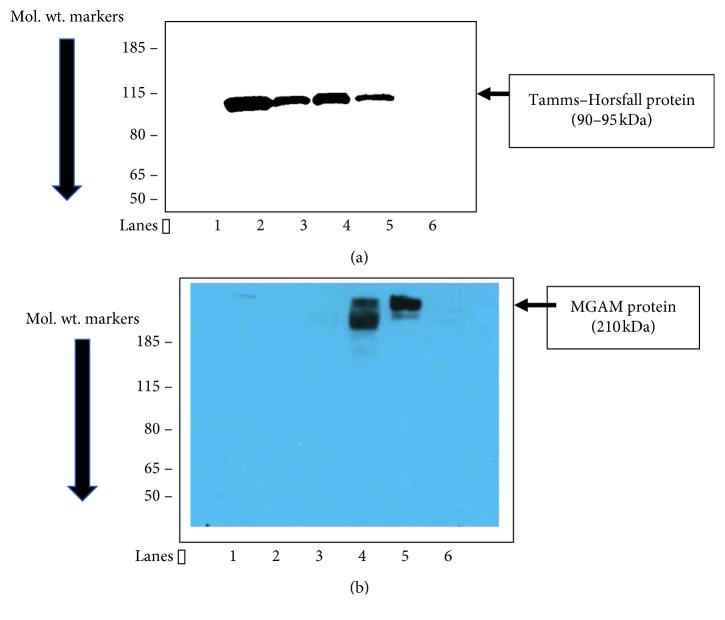
(a, b) Immunoblotting of cirrhosis-AKI urine exosomes for maltase-glucoamylase (MGAM). (a) Tamms–Horsfall protein blot for exosome loading control. (b) MGAM protein blot. Lanes 1 and 6: blank, lanes 2 and 3: normal control (noncirrhotic) urine exosome protein, and lanes 4 and 5: cirrhotic subjects with ascites and kidney injury.

The proteomic data showed a higher concentration of MGAM in the urine exosomes of decompensated cirrhotic patients, with and without kidney injury (groups 2 and 3). This was the single most discriminating protein among all the four groups with a VIP score of 4.35 (Figure 3). Confirmatory western blotting of these exosomes demonstrated detectable protein only in cirrhotic patients with kidney injury (group 3) (Figure 4).

## 4. Discussion

We conducted a proteomic analysis of the urinary exosome content from patients with compensated cirrhosis, decompensated cirrhosis, and decompensated cirrhosis with AKI and compared them to healthy controls. The proteomic analysis of urinary exosomes in cirrhotic patients identified several potentially important biomarkers of kidney injury, most notably MGAM, a bifunctional enzyme. We found the highest concentrations of MGAM in the urinary exosomes of the patients with cirrhosis and AKI. Furthermore, MGAM was increased in patients with cirrhosis but not to the extent as those with AKI. MGAM was absent in the healthy control group, highlighting its potential role for a biomarker of critical illness.

This study has several other important findings. First, to our knowledge, this is the first report on descriptive analysis of urinary exosome protein content in well-characterized cirrhotic subjects. Second, this is the first study to report increased tubular epithelial disaccharidase in the cirrhotic-kidney injury paradigm. The urine exosome proteomic data from the 4 different groups showed MGAM upregulation in the cirrhosis AKI group to be robust and consistent. Maltase is the major disaccharidase in renal brush border membranes [26, 27], but the precise function of this enzyme is not clearly elucidated; however, a possible role for the related disaccharidases, sucrase-isomaltase, and trehalase in sugar transport has been postulated [28]. In ten mammalian species, disaccharides related to MGAM have been found in renal brush border [27]. Farquhar and colleagues have demonstrated that maltase is present in the microvilli of the proximal convoluted tubule, perhaps functioning in glucose reabsorption and transport; and this absorptive capacity decreases towards more distal portions of the nephron [29]. MGAM was shown to be present in exosomes and microparticles in a mouse model of nonalcoholic steatohepatitis (NASH) [30], a common cause of cirrhosis. Since cirrhotic patients have several underlying conditions that contribute to a decrease in Scr, the detection of AKI is problematic. Additionally, the etiology of injury is often unknown, and differentiating between hepatorenal syndrome and other etiologies of AKI is difficult. Furthermore, the pathophysiology of decompensated cirrhosis with AKI is unclear as it may reflect hemodynamic changes or inflammatory mediators in the case of acute on chronic liver failure [31]. We reasoned that the structural and functional changes that cirrhosis and portal hypertension bring about in kidney function vary in severity according to the severity of cirrhosis. These differences between the compensated and decompensated liver disease with kidney injury can be understood by studying the downstream products of the kidney, such as urine. Given the nephron cell-state-specific cargo of the urinary exosome, we hypothesized that urine exosome analysis holds key information that is relevant to the differentiation of AKI in cirrhosis. This report is the first step towards testing this hypothesis.

This study design had several limitations. First, the number of subjects analyzed per group is small. A larger sample size may have increased the robustness of these data further, and additional proteins may have reached the threshold of an acceptable FDR of <10%. However, despite this small sample, these data demonstrating MGAM as a unique exosomal protein in cirrhotic patients with AKI is robust. Second, we used 1d gel electrophoresis to resolve the exosome proteins prior to LC/MS-MS analysis that resulted in identification of 1572 proteins overall. If we had conducted direct exosome protein trypsinization instead of following this method, perhaps the number of identified proteins might have increased. In our experience, exosomes are packaged with nonfull length peptides from proteolytic action as well as endogenous peptides and may have confounded the analysis. By following the gel electrophoresis method, we ensured that we only compared full-length protein differences between groups.

In summary, we have characterized urine exosome protein differences in healthy controls and compensated and decompensated cirrhotic subjects with and without AKI by proteomic methods. Work from the Knepper group shows that many important renal proteins (e.g., aquaporins, polycystins, and podocin) are shed in the urine exosome [32, 33]. Our current report adds MGAM to this group of functionally important renal proteins identified in urine exosomes. Our findings suggest that MGAM may differentiate proximal tubular injury from other types of AKI in cirrhotic patients. However, the clinical significance of MGAM upregulation in cirrhosis subjects with AKI needs to be established in future studies.

## Figures and Tables

**Table 1:** Study subject demographics.

Variable	Group 0, *N*=6	Group 1, *N*=6	Group 2, *N*=6	Group 3, *N*=6
Age (years)	28.7 ± 5.3	62.6 ± 7.2	58.5 ± 7.8	49.9 ± 7.5
Gender, M	6	6	6	6
Race/ethnicity				
White	2	3	2	1
Black	0	0	1	0
Asian	3	2	2	0
Hispanic	1	0	1	5
Others	0	1	0	0
Height (cm)	174.8 ± 4.9	163.8 ± 11	168.4 ± 8.7	170.5 ± 10.2
Weight (kg)	83.23 ± 11.8	72.6 ± 15.9	78.2 ± 15.8	86.8 ± 23.7
BMI (kg/m^2^)	26.3 ± 4.1	27 ± 5.2	27.5 ± 4.6	29.6 ± 5.8
Etiology of liver disease	NA			
Hepatitis C		5	4	4
Alcoholic cirrhosis		0	1	2
Hepatitis C and alcohol		1	1	0
NASH		0	0	0
Hepatitis B		0	0	0
Autoimmune		0	0	0
Other		0	0	0
Child-turcotte-pugh score	NA	5.2 ± 0.4	8.3 ± 1.8	10.1 ± 1.9
Child-turcotte-pugh class	NA			
A		5	4	0
B		0	2	3
C		0	0	3
Unable to classify		1	0	0
MELD score	NA	7.5 ± 1.8	11.7 ± 4.3	21.2 ± 8.5
Cryoglobinemia	NA	1	2	2
Spleen (cm)	NA	12.2 ± 2.4	14.8 ± 4.1	16 ± 3.7
Diabetes	0	1	3	2
Hypertension	0	3	2	1
Systolic blood pressure (mmHg)	128.7 ± 8.3	127.6 ± 20.8	123.7 ± 17.3	110.1 ± 15.3
Diastolic blood pressure (mmHg)	72 ± 9.5	78 ± 8.2	74.4 ± 8	65.4 ± 9.7
Scr within 30 days (mg/dL)	0.84 ± 0.1	0.74 ± 0.14	0.74 ± 0.23	2.1 ± 1.4
Platelets (10^9^ per liter)	NA	146.4 ± 88.1	107.3 ± 67.8	85.2 ± 51.5
History of varices	NA	1	4	2
History of variceal bleeding	NA	0	1	0
History of TIPS	NA	0	2	1
History of HRS				
Type 1	NA	0	0	0
Type 2	NA	0	0	0
History of SBP	NA	0	3	5
History of ascites	NA	0	6	6
History of transplant				
Liver	NA	0	0	0
Kidney	NA	0	0	0

**Table 2:** Top discriminating proteins for AKI with false discovery rate < 10%.

GI number	Protein name	VIP score
4758712	Maltase-glucoamylase (*Homo sapiens*)	4.3529
5802984	UDP-GlcNAc:betaGal beta-1,3-N-acetylglucosaminyltransferase 1 (*Homo sapiens*)	3.5869
119703753	Keratin 6B (*Homo sapiens*)	3.5388
5031809	Immunoglobulin superfamily containing leucine-rich repeat (*Homo sapiens*)	3.3817
4506153	Prostasin preproprotein (*Homo sapiens*)	3.3263
88702793	Slit-like 2 (*Homo sapiens*)	3.2463
24234699	Keratin 19 (*Homo sapiens*)	3.0505
4506121	Protein Z, vitamin K-dependent plasma glycoprotein (*Homo sapiens*)	2.9283
9966777	Resistin (*Homo sapiens*)	2.8229
4503491	Epidermal growth factor (beta-urogastrone) (*Homo sapiens*)	2.715
89357932	Keratin 5b (*Homo sapiens*)	2.5725
156523970	Alpha-2-HS-glycoprotein (*Homo sapiens*)	2.4263
4557391	Complement component 8, beta-polypeptide preproprotein (*Homo sapiens*)	2.0704

## Data Availability

The clinical and proteomic data used to support the findings of this study are restricted by the UCSD institutional review board in order to protect patient privacy. The data are available from Dr. Linda Awdishu (lawdishu@ucsd.edu) for researchers who meet the criteria for access to confidential data.
